# Clutch may predict growth of hatchling Burmese pythons better than food availability or sex

**DOI:** 10.1242/bio.058739

**Published:** 2021-11-19

**Authors:** Jillian M. Josimovich, Bryan G. Falk, Alejandro Grajal-Puche, Emma B. Hanslowe, Ian A. Bartoszek, Robert N. Reed, Andrea F. Currylow

**Affiliations:** 1 U.S. Geological Survey, Fort Collins Science Center - South Florida Field Station, 40001 SR 9336, Homestead, FL 33034, USA; 2 Conservancy of Southwest Florida, Naples, FL 34102, USA; 3 U.S. Geological Survey, Fort Collins Science Center, Fort Collins, CO 80526, USA

**Keywords:** Morphology, Energetics, Phenotypic variation, Resource acquisition, Invasive species, Reptile

## Abstract

Identifying which environmental and genetic factors affect growth pattern phenotypes can help biologists predict how organisms distribute finite energy resources in response to varying environmental conditions and physiological states. This information may be useful for monitoring and managing populations of cryptic, endangered, and invasive species. Consequently, we assessed the effects of food availability, clutch, and sex on the growth of invasive Burmese pythons (*Python bivittatus* Kuhl) from the Greater Everglades Ecosystem in Florida, USA. Though little is known from the wild, Burmese pythons have been physiological model organisms for decades, with most experimental research sourcing individuals from the pet trade. Here, we used 60 hatchlings collected as eggs from the nests of two wild pythons, assigned them to High or Low feeding treatments, and monitored growth and meal consumption for 12 weeks, a period when pythons are thought to grow very rapidly. None of the 30 hatchlings that were offered food prior to their fourth week post-hatching consumed it, presumably because they were relying on internal yolk stores. Although only two clutches were used in the experiment, we found that nearly all phenotypic variation was explained by clutch rather than feeding treatment or sex. Hatchlings from clutch 1 (C1) grew faster and were longer, heavier, in better body condition, ate more frequently, and were bolder than hatchlings from clutch 2 (C2), regardless of food availability. On average, C1 and C2 hatchling snout-vent length (SVL) and weight grew 0.15 cm d^−1^ and 0.10 cm d^−1^, and 0.20 g d^−1^ and 0.03 g d^−1^, respectively. Additional research may be warranted to determine whether these effects remain with larger clutch sample sizes and to identify the underlying mechanisms and fitness implications of this variation to help inform risk assessments and management.

This article has an associated First Person interview with the first author of the paper.

## Abstract (precis)

**Summary:** Hatchling pythons from an invasive population displayed substantial phenotypic variation in morphometrics, growth rates, and behaviors. This information may be useful for managing populations of cryptic, endangered, and invasive species.

## INTRODUCTION

Evolutionary trade-offs in the allocation of energy and resources affect fundamental life-history characteristics such as reproduction, growth, and survival (Lim et al., 2014). Organisms must allocate the finite resources available within their environment across these life-history characteristics to maximize fitness, but resource availability fluctuates by season and over years. Therefore, how individual organisms acquire and apportion resources results in intraspecific heterogeneity in growth and survival of their offspring (van Noordwijk and de Jong, 1986).

Among reptiles, resource availability often correlates with morphological development, as individuals with greater food intake can allocate those caloric resources to growing larger and faster relative to individuals with less access to food (e.g. snakes, turtles, lizards; Bonnet et al., 2001; Cox and Secor, 2007; Cox et al., 2008; Forsman, 1991; Lee et al., 2007; Madsen and Shine, 2000, 2002; Taylor and Denardo, 2005). Similarly, resource availability affects how reptiles exhibit trade-offs between physiological costs like fixed structural growth (e.g. body length) and energy storage (e.g. body condition). For instance, it may be favorable to allocate more energy to fixed structural growth when food is abundant to reach body size thresholds that enable early reproductive maturity or reduce predation risk, whereas it may be better to store energy in adipose tissue when food is scarce to reduce starvation risk (Arendt, 1997; Forsman and Lindell, 1991). Differences in local habitats and stochastic weather conditions affect prey abundance, which can profoundly influence the growth trajectories of local ecotypes and cohorts such that they experience life-long impacts (e.g. Madsen and Shine, 1998; Palacios et al., 2012).

However, resource trade-off ‘decisions’ begin even before the individuals they impact. Variation in parental phenotype and genotype (i.e. clutch effects; Webb et al., 2001) influences life history trade-offs between offspring quantity and quality, which is reflected in the considerable differences in morphologies, growth patterns, and behavioral phenotypes that can occur among individual young (Bergeron et al., 2011; Cam et al., 2013), as well as entire clutches. In oviparous reptiles, variation in maternal resource provisioning during any single reproductive attempt can affect offspring quantity (clutch size) and quality (hatchling size; Brown and Shine, 2009; Madsen and Shine, 1998). These maternal effects on hatchling phenotypes are multifaceted (Madsen and Shine, 1998; Sinervo et al., 1992). A female's investment in the quantity and composition of the yolk stores she allocates to each ovum will fuel pre- and post-hatching growth (Warner and Andrews, 2002), mediate hatchling behaviors through the steroid hormones deposited in the yolk, and alter immune function (Bowden et al., 2000; Warner et al., 2009). Females select nesting sites to optimize the incubation environment so that it is most conducive to certain developmental growth patterns (Burger et al., 1987; Shine et al., 1997). Mothers can also affect the genetic quality of their offspring through mate selection and sperm competition (Madsen et al., 1992; Shine, 2003), which can directly influence hatchling growth rates via genes that enable hatchlings to grow faster (e.g. Olsson et al., 1996a).

Researchers may also need to consider how heterogeneous growth patterns vary across ontogeny and between sexes to understand their fitness consequences. Many species of reptiles exhibit sexual size dimorphisms (SSD) from when they are neonates through adulthood, which can be expressed as significantly different growth patterns and morphologies between the sexes (Cox et al., 2007; King et al., 1999). Sexual size dimorphism may reflect that body size thresholds appear to be more important for inducing reproductive development than age-specific timing in some reptiles (Congdon and van Loben Sels, 1993; Ford and Seigel, 1994; Shine, 1990; Taylor and Denardo, 2005). Many reptile species exhibit size-limited fecundity whereby growth patterns directly impact fitness by promoting body sizes that increase potential for greater quantities and/or qualities of offspring (Armstrong et al., 2018; Darwin, 1871; Du et al., 2005; Qualls and Shine, 1995). Natural and sexual selection may drive females to grow larger than males to increase the number or quality of eggs they can produce (Cox et al., 2007; Shine, 2003, 1994; Shine et al., 2006). Alternatively, adult males may be larger than females to increase mating success or because reproductive costs constrain female body sizes (Cox et al., 2007; Pincheira-Donoso and Hunt, 2017; Shine, 1994, 2003; Taylor and Denardo, 2005). Furthermore, intraspecific variation in SSD can be driven across populations by interactions between growth, body size, and environmental factors such as food availability (Cox et al., 2007; Madsen and Shine, 1993; Wikelski and Trillmich, 1997).

Understanding how intraspecific growth is affected by environmental variation versus genetic variation can help biologists predict how populations will respond to different environmental conditions and physiological states. Model organisms for such investigations are those that have close or obligatory physiological ties to environmental conditions (such as ectotherms), are highly fecund, exhibit SSD, and are known to be tolerant of captive laboratory conditions. Burmese pythons (*Python bivittatus* Kuhl) are a good model for such studies because they are one of the largest snake species in the world, can produce large clutch sizes (an invasive female from Florida can have as many as 87 eggs; Krysko et al., 2012), exhibit indeterminate growth, are able to mature rapidly (i.e. can reach reproductive size within 1 year in captivity; Ross and Marzec, 1990; Willson et al., 2014), and display dramatic SSD with females growing much longer and heavier than males (Reed and Rodda, 2009, and references therein). The Burmese python also has a history as a model organism in studies of extreme phenotypic plasticity and physiological regulation of digestion (e.g. Secor, 2008; Secor and Diamond, 1998; Starck and Beese, 2001), cardiac function (e.g. Riquelme et al., 2011; Slay et al., 2014), and metabolism (e.g. Castoe et al., 2013; Secor and Diamond, 1997; Starck et al., 2004). Additionally, the biology of Burmese pythons is of interest because, though they are endangered in parts of their native range (Jiang et al., 2016), they have proliferated in and caused severe damage to the Greater Everglades Ecosystem as an invasive species (e.g. Dorcas et al., 2012). Researchers and land managers are keen to better understand python biology and ecology that may lead to more effective methods of population management.

Very little is known about the life history characteristics (e.g. growth or survival rates) or behavior of Burmese pythons in the wild, both in their native and introduced ranges. Burmese pythons are cryptic (i.e. detection probabilities are <5%; Nafus et al., 2020), occupy vast ecosystems that are very challenging to traverse (e.g. the Greater Everglades Ecosystem), and can survive in a broad range of environmental conditions (Bartoszek et al., 2018; Hart et al., 2012), thus making it a difficult species to study. Less life history information is known about free-ranging hatchling and juvenile pythons relative to adults. Consequently, almost all the experimental research conducted with Burmese pythons to date has been in captivity using animals sourced from the pet trade. Using captive animals is not ideal for understanding wild python ecology, as they are subject to artificial selection that could lead to lineages with traits and genotypes that are not representative of wild populations (Archard and Braithwaite, 2010). Thus, studies that incorporate pythons from wild populations may better reflect natural community dynamics.

In 2015, we had the rare opportunity to obtain several large clutches of eggs laid in the wild by free-ranging Burmese pythons that were a byproduct of a long-term radio-telemetry study examining their spatial ecology in southwestern Florida, USA (Bartoszek et al., 2021). We used the hatchlings from these clutches to conduct an experiment in captivity to determine if food availability, clutch, and sex might influence their growth patterns. The purpose of this study was to use Burmese pythons from an invasive population as a model to better understand how phenotypic variation in growth is modulated in the wild in response to varying environmental or physiological states, as this could help us better understand reptilian energetics and have implications for invasive species management.

Specifically, we wanted to know if food availability, clutch, or sex could explain phenotypic variation in hatchling body condition, length, weight, and growth rate by provisioning hatchling treatment groups with one of two differing quantities of food (High or Low) and observing their resource allocation strategies over time. We predicted that food availability would best explain any phenotypic variation we might find given that it correlates with morphological development in many other reptilian taxa (e.g. snakes, turtles, lizards; Bonnet et al., 2001; Cox and Secor, 2007; Cox et al., 2008; Forsman, 1991; Lee et al., 2007; Madsen and Shine, 2000, 2002; Taylor and Denardo, 2005). While not original components of our study, we also had the opportunity to evaluate resource acquisition through the hatchlings' behavioral responses to food availability (did or did not eat) and to investigate if the hatchlings' tendencies to consume food could be explained by feeding behavior (bold, neutral, or shy). These personality traits can help us interpret individual behavioral responses that may contribute to fitness tradeoffs (e.g. growth-mortality; Stamps, 2007). To our knowledge, we are the first to evaluate the growth patterns and feeding behaviors of hatchling Burmese pythons sourced from an invasive population, which may better reflect their life history characteristics in the wild.

## RESULTS

Initial measurements at the start of the experiment (i.e. week 1) showed no morphometric differences among study animals within either feeding treatment or by sex, but there were significant differences in body condition index (BCI), length, and weight between hatchlings from different clutches (see below). None of the 30 hatchlings offered a mouse during the yolk phase consumed the provided food. Sixteen of the 60 study animals [four clutch 1 (hereafter, C1) and 12 clutch 2 (hereafter, C2), eight male and eight female, and ten High and six Low] refused all food throughout the study. Two of those (two females from C2 assigned to High) were humanely euthanized prior to the end of the study due to particularly poor body condition, and we did not use their associated data in analyses. Additionally, a week 1 weight measurement was not collected for one C2 male in the Low feeding treatment, so this snake was excluded from analyses that necessitated a week 1 weight.

### Morphological growth comparisons

**Fig. 1.**
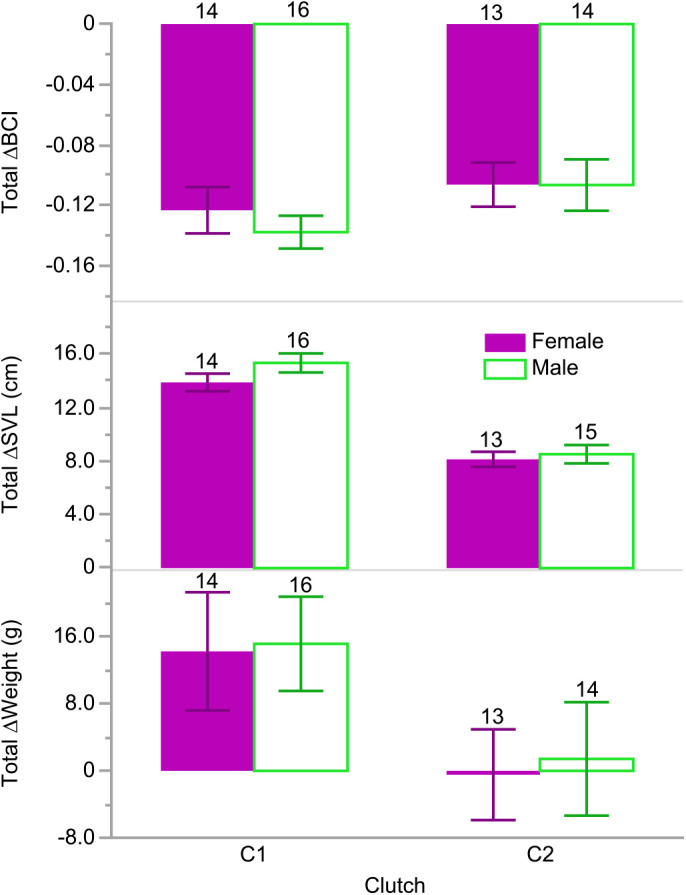
**Total morphological changes in body condition indices (ΔBCI), snout-vent lengths (ΔSVL), and weights (Δweight) of hatchling *Python bivittatus* Kuhl in each clutch by sex [female (solid purple bars) and male (hollow green bars)] over 12 weeks post-hatching.** Error bars are standard error of the means (s.e.m.). Sample sizes for each group are indicated above the error bars. Note: the snakes were sustained by internal yolk stores during the first 4 weeks post-hatching.

**Fig. 2.**
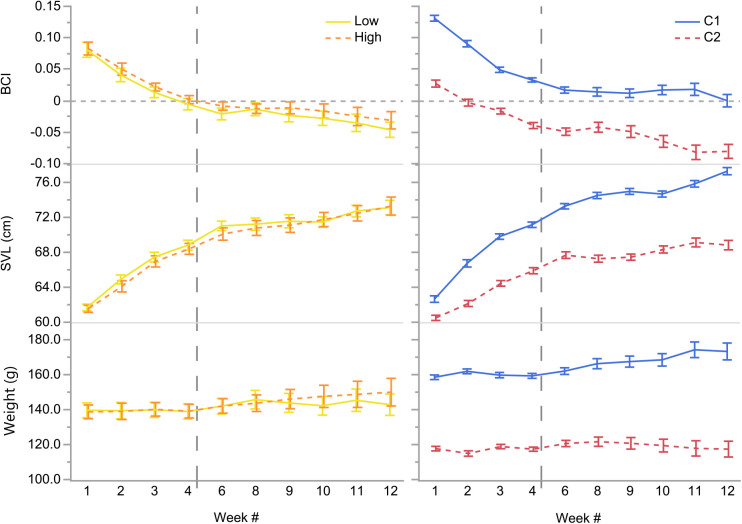
**Mean BCI, SVL, and weights each week by feeding treatment [Low (solid yellow line) and High (dashed orange line); left] and clutch [C1 (solid blue line) and C2 (dashed red line); right] of hatchling *Python bivittatus* Kuhl.** The horizontal dashed grey line represents the predicted mean body condition, where more robust conditions are positive numbers above, and poorer conditions are negative below. The vertical dashed grey line represents the behavioral phase change from the Yolk Phase (when the pythons were purportedly sustained by internal yolk stores) to the Eating Phase (when pythons began eating the offered food) at the end of week 4. Error bars are standard error of the means (s.e.m.). Data from 58 hatchlings are represented in this figure over time.

Total change in snout-vent length (ΔSVL) and total change in weight (Δweight) were independently correlated with clutch (*F*_1,56_=88.5074*, P*<0.0001 and *F*_1,55_=5.3413, *P*=0.0246, respectively), where hatchlings from C1 exhibited greater changes in SVL (14.6 cm versus 8.3 cm), and more total weight gain (14.8 g versus 0.5 g) than hatchlings from C2 (Fig. 1). Concordantly, clutch explained differences in weekly BCI, SVL, and weight throughout the project (all *P*<0.0001) where clutchmates grouped similarly despite feeding treatments (Fig. 2; Table S1). We found no morphometric differences in hatchlings assigned to the feeding treatments at the beginning or end of the experiment (i.e. weeks 1 and 12; all *P*>0.4; Table S1). However, there were differences in hatchling mean BCIs, SVLs, and weights between clutches at the beginning and end of the experiment (all *P*≤0.0066; Table S1). There were no weekly differences in BCI, SVL, or weight between hatchlings in different feeding treatments or over the 12 weeks. We found no sex differences in morphometrics during any week excepting week 5 when females were temporarily found to be slightly longer than males (72.5 versus 71.0 cm; *F*_1,28_=7.9784, *P*=0.0086). Additionally, we found no effect of feeding treatment or sex on any total Δmorph.

### Growth rates and resource allocation by behavioral phase

**Fig. 3.**
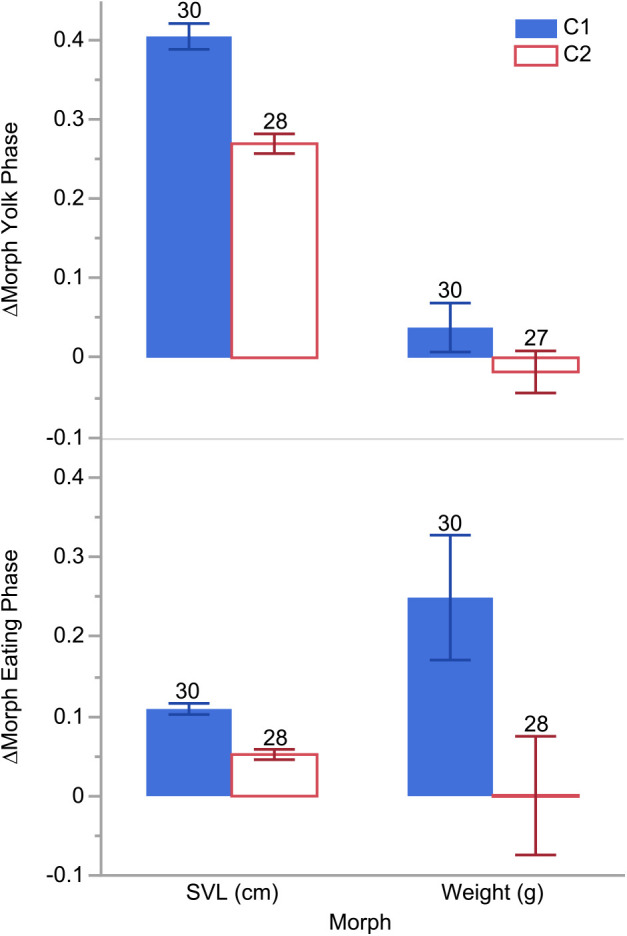
**Resource allocation bar plot of daily changes in morphologies measured in SVL and weights in hatchling *Python bivittatus* Kuhl from each clutch [C1 (solid blue bars) and C2 (hollow red bars)] by behavioral phase [yolk phase (top) and eating phase (bottom)] of the study.** Error bars are 95% confidence interval (CI). Sample sizes for each group are indicated above the error bars.

We detected no overall differences between feeding treatments or sex in the rate of morphometric change per day (growth rates in Δmorph d^−1^). However, ΔSVL d^−1^ growth rates differed between hatchlings in the different clutches (*F*_1,56_=43.1933, *P*<0.0001), but ΔBCI d^−1^ and Δweights d^−1^ did not (Table 1). We found clutch effects in both behavioral phases for ΔBCI d^−1^ and ΔSVL d^−1^ but only during the eating phase for Δweight d^−1^. During the yolk phase, hatchlings in C1 had a more negative ΔBCI d^−1^ (−0.005 versus −0.003 d^−1^; *F*_1,56_=44.136, *P*<0.0001) and a greater increase in ΔSVL d^−1^ (0.40 versus 0.27 cm d^−1^; *F*_1,56_=42.6898, *P*<0.0001) than hatchlings in C2, but there were no detectable weight differences by clutch (Fig. 3). Interestingly, sex effects were detectable in the yolk phase for Δweights d^−1^ where females decreased in weight at a rate of −0.05 g d^−1^ while males increased by 0.6 g d^−1^ (*F*_1,56_=8.8615, *P*=0.0043). We detected no other growth rate differences between sexes. During the eating phase, hatchlings from C1 grew longer and heavier more quickly than those from C2 (ΔSVL d^−1^=0.11 versus 0.05 cm d^−1^; *F*_1,56_=35.8540, *P*<0.0001; Δweight d^−1^=0.25 versus 0.00 g d^−1^; *F*_1,56_=5.2536, *P*=0.0257; Fig. 3).

**Table 1.**
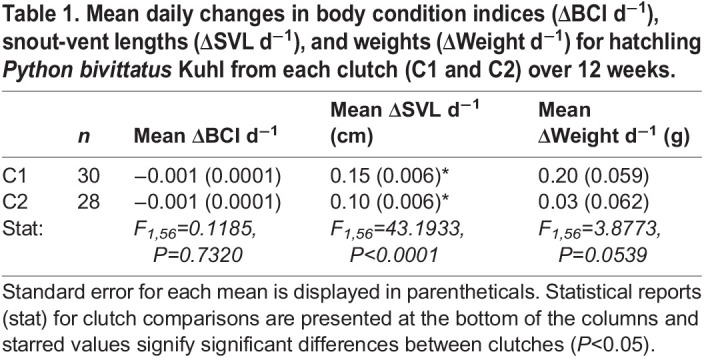
Mean daily changes in body condition indices (ΔBCI d^−1^), snout-vent lengths (ΔSVL d^−1^), and weights (ΔWeight d^−1^) for hatchling *Python bivittatus* Kuhl from each clutch (C1 and C2) over 12 weeks.

### Behavioral comparisons

All animals offered meals during the yolk phase refused to eat. During the eating phase, we found that only 31.0% of the 58 study animals used in analyses ate every time food was offered, 12.1% ate only once regardless of food availability, and 24.1% refused food altogether. Of the animals that refused all food during the study, four were from C1 (13.3% of C1) and ten were from C2 (35.7% of C2). We found that 7.1% of study animals in the High group ate all nine mice offered over the eating phase whereas 53.3% of the animals in the Low group ate all three mice offered over the 3-week intervals.

**Fig. 4.**
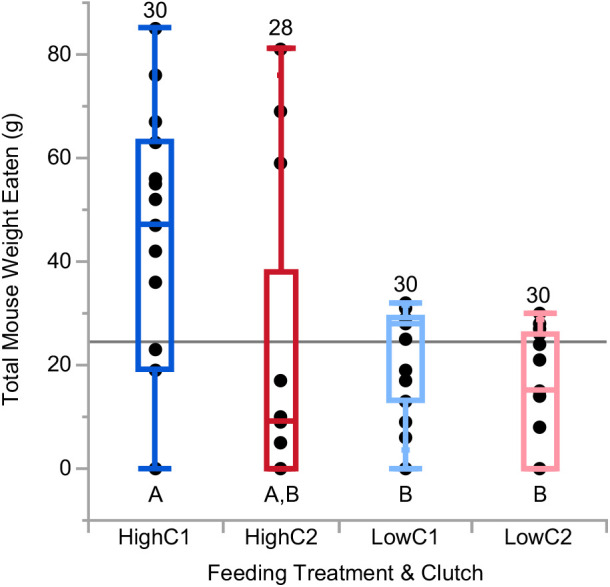
**Quantile boxplots (median, interquartile range, and min/max values) of total grams of mouse eaten by feeding treatment [High (dark colors) and Low (light colors)] and clutch [C1 (blue boxplots) and C2 (red boxplots)] of hatchling *Python bivittatus* Kuhl.** Boxplots that share a letter are not significantly different (*P*>0.05). The solid grey horizontal line represents overall mean grams consumed (24.5 g). Note: 14 of the final 58 study animals refused food throughout the study, and they are included in the sample sizes for each group which are indicated above each boxplot (three HighC1, five HighC2, one LowC1, five LowC2).

Total grams of mouse eaten was not associated with sex but was predicted by both feeding treatment (*F*_1,56_=5.1826, *P*=0.0267) and clutch effect (*F*_1,56_=6.0412, *P*=0.0171), where High versus Low treatments consumed 31.4 g (s.e.=4.2) versus 18.0 g (s.e.=4.1), and individuals from C1 consumed more (31.4 g, s.e.=4.1) than those from C2 (17.1 g, s.e.=4.2; Fig. 4).

We found differences in feeding propensity between treatments and clutches, but not between sexes. The probability of eating was higher among individuals in the Low feeding treatment (ate 67% of the time food was offered) than those in the High feeding treatment (37% of the time food was offered; χ^2^_(1, *n*=284)_=22.659, *P*<0.0001) even though the total meals eaten was more than double in the High versus Low feeding treatments (averaging 3.1 versus 1.5 meals eaten; *F*_1,56_=7.9616, *P*=0.0066). Individuals from C1 ate more of the total number of meals offered than did those from C2 (χ^2^_(1, *n*=284)_=24.623, *P*<0.0001), averaging twice as many total meals (averaging 3.0 versus 1.5 total meals eaten; *F*_1,56_=6.8566, *P*=0.0113).

**Fig. 5.**
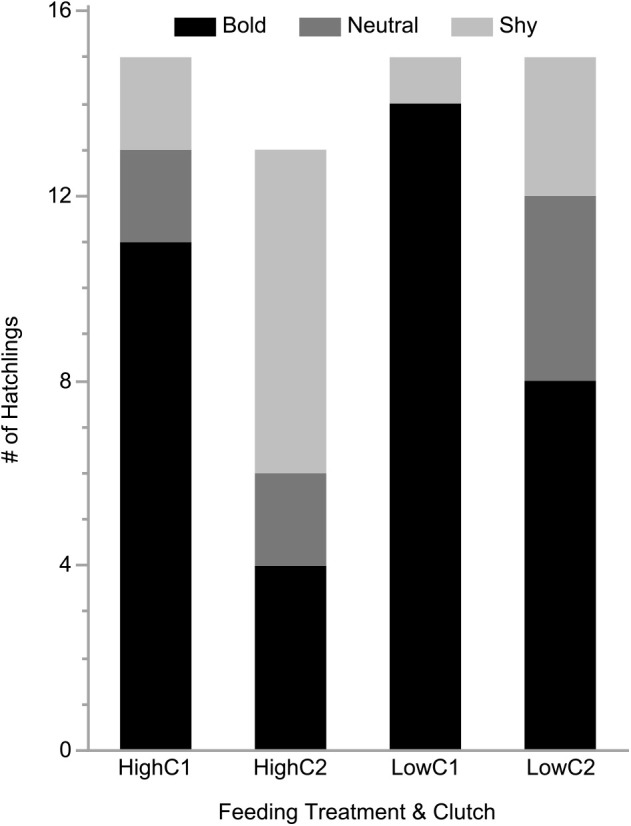
Total number of hatchling *Python bivittatus* Kuhl exhibiting bold (e.g. aggressive/immediately attempted to feed; black sections), neutral (e.g. neither aggressive nor shy; dark grey sections), or shy (e.g. retreated from prey; light grey sections) feeding behaviors by feeding treatment (High and Low) and clutch (C1 and C2) during the final week that all hatchlings were offered food (week 10 of the experiment).

The feeding behaviors recorded in week 10 were not distinguishable by feeding treatment or sex but revealed differences between clutches. Individuals from C1 were more likely to be ‘bold’, whereas those in C2 were more often ‘shy’ or ‘neutral’ in response to food (χ^2^_(2, *n*=58)_=10.280, *P*=0.0059; Fig. 5).

## DISCUSSION

We predicted that food availability would best explain any phenotypic variation we might find among the Burmese python hatchlings given that it correlates with morphological development in many other reptilian taxa (e.g. snakes, turtles, lizards; Bonnet et al., 2001; Cox and Secor, 2007; Cox et al., 2008; Forsman, 1991; Lee et al., 2007; Madsen and Shine, 2000, 2002; Taylor and Denardo, 2005). Surprisingly, we found that clutch was a better predictor of variation in hatchling python growth and size than feeding treatment or sex (Fig. 1 and Fig. 2). Hatchlings from C1 grew faster and were longer (Table 1, Fig. 3), heavier (Fig. 3), in better body condition, ate more (Fig. 4), and were bolder than hatchlings from C2 (Fig. 5), regardless of food availability.

While we designed the study with balanced hatchling sample sizes across the feeding treatments and sexes, the unexpected morphometric, growth, and behavioral variation we observed among C1 and C2 hatchlings throughout the experiment may have resulted from our small sample size (*n*=2 clutches). Wild Burmese python clutches are difficult to obtain given how cryptic the species is (detectability <5%; Nafus et al., 2020), and the clutch differences we observed may not have been detectable or significant within the overall population if we had studied hatchlings from more clutches. Additional research using more clutches is needed to help us understand whether the clutch differences we detected reflect true variation within the wild population, and, if so, which clutch effects might explain this variation (e.g. maternal body size, nest site condition, etc.). There are many factors that could be explored including parental genetic quality (Olsson et al., 1996a), maternal body size (Nafus et al., 2015), maternal stress levels (Warner et al., 2009), maternal yolk investment (Warner and Lovern, 2014), nest location and microclimate (Brown and Shine, 2004; Shine et al., 1997), and so on. Below, we discuss the patterns we observed in more detail to promote additional research avenues, along with some of the mechanisms that may be driving these patterns and their implications for python fitness in the wild.

Across all the study snakes, the fastest SVL growth rates and BCI declines were during the yolk phase when the hatchlings were relying entirely upon their yolk energy stores (Fig. 2). Madsen and Shine (2002) concluded that hatchling water pythons (*Liasis fuscus* Peters) initially grew rapidly in length by depleting their energy stores, thereby trading higher body condition (i.e. storage) for increased length. They hypothesized that water pythons were growing in length to be able to feed on larger and more abundant prey items (i.e. adult dusky rats, *Rattus colletti* Thomas; Madsen and Shine, 2002). In the present study, Burmese python hatchlings may have exhibited a similar fixed resource allocation strategy by prioritizing early-life length growth over fat storage and higher body condition to improve post-hatching survival and fitness. The advantage might be that longer hatchlings are better suited to defend themselves against predators or to subdue larger prey, representing a trade-off between energy storage and mortality risk through starvation or predation.

Alternatively, there may be selection pressure for hatchlings to grow longer so that they can reach sexual maturity earlier. Burmese pythons can mature at relatively short lengths (i.e. ≤185 cm SVL; Reed and Rodda, 2009; Willson et al., 2014), and size appears to determine timing of sexual maturity more than age in many reptiles (e.g. colubrids, viperids, testudines; Congdon and van Loben Sels, 1993; Ford and Seigel, 1994; Shine, 1990; Taylor and Denardo, 2005). As adults, pythons exhibit SSD with females reaching much larger sizes than males (Reed and Rodda, 2009, and references therein), so we expect that sex effects on growth would be detected over longer time frames than this study. Indeed, Taggart et al. (2021) found that captive-raised Burmese python growth rates began differentiating after the snakes were 100 days old, and our study was concluded before the snakes reached that age. The only sex effect we found was that females decreased in weight while males grew heavier during the yolk phase, which, as far as we can deduce, can only be attributable to differences in water consumption. There were clutch differences in the growth-storage trade-off during the yolk phase that did not persist into the eating phase for both clutches. The C1 hatchlings devoted more energy resources to length growth and storage throughout the experiment than C2 hatchlings, especially in the eating phase, when they gained weight (0.25 g d^−1^ opposed to 0.00 g d^−1^) and over twice as much length per day as C2 hatchlings (0.11 cm d^−1^ versus 0.05 cm d^−1^; Fig. 3).

Although both clutches were kept under identical conditions, we found that C1 hatchlings had a greater propensity to feed than C2 hatchlings in that they ate more than double the number of meals over the course of the experiment (3.1 versus 1.5 meals eaten) and were more likely to behave boldly near the end of the experiment (week 10; Fig. 5). Yet, 24.1% of all hatchlings refused food altogether throughout the experiment. The hatchlings that refused all food may have had enough energy from their yolk stores to sustain them, and they might have begun eating if the experiment had gone on longer. The age of first feeding does not appear to be well documented for reptiles in the primary literature, so there may be more intraspecific variation in the onset of feeding among wild reptiles than has been realized. Alternatively, the fasting behavior we observed could be an artifact of captivity. Reptile behavior and physiology are closely tied to environmental cues, so conditions experienced in captivity may significantly alter the physical, behavioral, and endocrine phenotypes that are expressed (e.g. Currylow et al., 2017b). We offered the hatchlings frozen/thawed rodents averaging 9 g, which is similar in weight to some of the smaller prey items observed in invasive, wild python diets such as North American least shrews (*Cryptotis parva* Say; average weight=5 g; Wilman et al., 2014) and neonate hispid cotton rats (*Sigmodon hispidus* Say and Ord; weight range=5–8 g; Randolph et al., 1977; Christina M. Romagosa, University of Florida, written communication, 2020). Though the feeder rodents we used were frozen/thawed, snakes from both feeding treatments, clutches, and sexes recognized them as food and consumed them. Furthermore, some of the smallest hatchlings in our clutches ate at the beginning of the eating phase and some of the largest hatchlings ate throughout the experiment, which suggests that these meal sizes were appropriate for the hatchlings to ingest, even though they are capable of consuming larger prey.

The feeding frequencies and typical prey sizes of wild Burmese python hatchlings relative to their body weights remain unknown. Wild hatchlings may naturally forego feeding opportunities at the expense of growth and energy storage in certain situations. Many reptile species express innate antipredator responses immediately after birth or hatching (e.g. Baxter-Gilbert et al., 2018; Herzog and Burghardt, 1986) when they are vulnerable. Inexperienced animals may perceive exaggerated predation risks, even to the extent that they compromise their own fitness (Lima, 1998; Lima and Dill, 1990). Foraging opportunities often have associated mortality risks through increased exposure time to predators (Lima and Dill, 1990), and snakes may be more susceptible during times when they are incapacitating, handling, or digesting prey (Cruz-Neto et al., 1999; Garland and Arnold, 1983; Nielsen et al., 2011; Siers et al., 2018). Yet some research with snake neonates has indicated that larger individuals are bolder, which reflects inherent differences in behavioral traits between hatchlings of different sizes (Mayer et al., 2016). Consequently, the variation in hatchling feeding responses we observed across clutches may indicate differences in inherent anti-predator decision making and behavioral traits. Smaller C2 hatchlings could have been making the trade-off to reduce mortality risk at the expense of growth and storage because they perceived feeding opportunities as considerably more risky on average than larger C1 hatchlings, which is concordant with prior studies investigating reptile clutch effects which found that they explained much of the variation in innate antipredator responses and body sizes (Baxter-Gilbert et al., 2018; Webb et al., 2001).

It is worth expounding more on why we did not find that food availability impacted growth patterns significantly when so many other studies have found relationships (Bonnet et al., 2001; Forsman and Lindell, 1991, 1996; Queral-Regil and King, 1998; Taylor and Denardo, 2005). As discussed above, the simplest explanation is that our study was limited in that we only compared hatchlings from two clutches, so it is entirely possible that food availability may have a more significant impact on hatchling growth patterns than clutch across the entire population. For instance, hatchlings in C1 High ate more grams of food than their clutchmates in C1 Low, a trend not seen in the C2 clutch (Fig. 4). Consequently, if the hatchlings from C2 had eaten more of the food that was available to them in both High and Low feeding treatments, we may have found that food availability would have had a more significant effect on growth than clutch. Alternatively, our data could be interpreted as evidence that the amount of food available to Burmese python hatchlings early in life does not impact their survival probabilities because body condition (which is often treated as a proxy for fitness; Jakob et al., 1996; Stevenson and Woods, 2006) did not have a relationship with food availability. Viewed in this way, hatchling pythons are well adapted to withstand hatching in sub-optimal locations with low prey availability or safety. Such an adaptation could have helped pythons establish throughout the Greater Everglades Ecosystem. However, some of our study pythons continued to decline in condition because they never overcame their apprehension to feeding. We are not the first to find that python hatchlings refuse food (e.g. Hart et al., 2012; Shine et al., 1997), sometimes until they perish (Secor, 1995). During a study of the digestive responses to feeding in captive-bred Burmese pythons, Secor (1995) found that three hatchlings from the same clutch could not be enticed to eat yet survived for up to 205 days without food. Though phenotypic variation in behaviors promotes population resilience, death from starvation before reproduction is certainly maladaptive. It is more likely that some clutches are inherently less likely to survive in the wild than others. The C2 nest had lower hatching success than the C1 nest (82% versus 100% of fertilized eggs hatched, respectively). Hatching success has been found to be positively correlated with survival (Madsen and Shine, 1998) and reproductive success (Olsson et al., 1996b, 2011) in other reptiles, which suggests that C2 hatchlings would have experienced lower fitness in the wild.

The phenotypic variation in growth patterns and feeding propensities we observed suggests that clutches may have experienced different fitness in the wild. Across many vertebrate taxa, large body sizes and faster growth rates can reduce susceptibility to mortality from predation, starvation, dehydration, and so on, translating to higher survival (e.g. lizards, snakes, tortoises, anurans, teleost fishes; Brown and Shine, 2004; Cabrera-Guzman et al., 2013; Ferguson and Fox, 1984; Nafus et al., 2015; Sogard, 1997). We hypothesize that the C2 hatchlings would have exhibited lower survival in the wild than C1 hatchlings given their lighter body weights, poorer body conditions, and slower growth rates.

We are aware of only three other studies on Burmese python growth that have reported on any of the morphometrics that we examined, and they all used captive-bred pythons (Cox and Secor, 2007; Starck and Beese, 2001; Taggart et al., 2021). Starck and Beese (2001) documented the average initial and final weights of pythons, but the study animals were considerably older than those used in the present experiment (approximately 9 months to 3 years of age). Taggart et al. (2021) and Cox and Secor (2007) were the only studies to evaluate how growth was influenced by any of the explanatory variables that we considered (sex, and feeding and clutch, respectively). As mentioned above, Taggart et al. (2021) documented that python growth was associated with sex, with the body weight of females increasing relative to males after approximately 100 days of age, when they were older than our hatchlings at the conclusion of this study. Cox and Secor (2007) used juvenile pythons to explore how meal size, clutch, and metabolism affected energy efficiencies and body weights, estimating that juveniles fed meals between 15–35% of their body mass grew between 1.7–6.2 g d^−1^. Contrary to our results, they found feeding effects; pythons that ate larger meals grew significantly heavier than those that ate smaller meals (Cox and Secor, 2007). In comparison, snakes in our study were younger, fed smaller meals, and exhibited much lower mean weight growth rates (C1=0.20 g d^−1^, C2=0.03 g d^−1^; Table 1). However, like Cox and Secor (2007), we found that clutch effects explained most of the observed phenotypic variation among hatchling phenotypes, consistent with literature on other reptiles (e.g. Madsen and Shine, 1998; Martínez-Caballero et al., 2017; Webb et al., 2001). Cox and Secor (2007) hypothesized that variation in digestive performance or metabolic expenditures could have been driving the interclutch differences, and they concluded that the clutch effects were more likely due to genetic rather than environmental factors. Alternatively, if persistent across the larger population, the clutch effects on growth and feeding behavior that we observed may have been determined by the hatchlings' maternal environments. Early developmental conditions and experiences can have strong immediate and long-lasting effects on offspring life-history traits (e.g. Brown and Shine, 2004; Holden et al., 2019; Shine et al., 1997). The eggs from C1 were incubated in the wild for a longer proportion of time than the C2 clutch, and the female pythons that laid these clutches were different sizes and chose different microhabitats for their nesting sites (C1 was laid on the ground surface in some grass, while C2 was laid in a burrow).

Future studies could more directly evaluate which maternal effects influence Burmese python fitness by investigating hatchling growth rates and survival in the wild by following animals through ontogeny, although it may not be possible to evaluate the influence of food availability in the field. Field experiments can be more informative than captive experiments if they can be designed to impose adequate experimental controls. However, implementing a field experiment similar to ours would be prohibitively difficult as it would necessitate artificially controlling free-ranging hatchlings' access to food or near consistent monitoring and documentation of a large number of subjects. Alternatively, other surrogate measures associated with fitness such as stress, locomotor performance, personality (e.g. Currylow et al., 2017a; Mutascio et al., 2017), body size, and timing of sexual maturity could be evaluated in captivity along with food availability.

Here we demonstrated that hatchling Burmese pythons sourced from an invasive population display significant phenotypic variation in morphometrics, growth rates, and behavior. Unexpectedly, we did not find differences between the target variables of food availability and sex, and instead found variation was clearly attributed to clutch. On average, we found that hatchlings from C1 maintained significantly heavier and longer bodies, higher BCIs, faster growth rates, greater feeding propensities, and bolder feeding behavior than hatchlings from C2. These results suggest that hatchlings can exhibit different growth patterns that, in conjunction with variable feeding behaviors, ultimately result in different survival rates. Focused studies could help determine if the differences we detected persist with larger clutch sample sizes and, if so, to provide insight into how clutch effects might drive interclutch fitness differences. Studies that can find innovative methods to assess the life-history strategies of cryptic species across all life stages may be useful for informing monitoring, management, and risk assessment efforts.

## MATERIALS AND METHODS

### Study animals

We acquired 136 python eggs from the nests of two wild Burmese pythons (C1, *n*=74; C2, *n*=62) located in Collier County, Florida, USA. There were 71 fertile eggs (19.4 kg) in C1 that all hatched, and there were 61 fertile eggs (12.5 kg) in C2, 50 of which hatched. The females that laid the clutches were part of a long-term radio-telemetry study examining adult Burmese python spatial ecology (Bartoszek et al., 2021). After laying eggs, the C1 female weighed 54.5 kg and was approximately 4.9 m total length, and the C2 female weighed 29.5 kg and was approximately 4.3 m total length. We removed the eggs from the wild nests in June 2015 and incubated them in the laboratory at 28–34°C (temperatures similar to those observed in wild and captive nests; Hutchison et al., 1966; Snow et al., 2010; Wolf et al., 2016) until hatching in July 2015. Because the animals in this study were sourced from the wild, we do not know precisely when the eggs were laid, but the C1 eggs hatched more quickly after they were moved to incubation than the C2 eggs (16 versus 41 days later, respectively). We probed each hatchling to determine sex (Schaefer, 1934), measured their SVL to the nearest 0.5 cm by carefully stretching them along a meter tape, and weighed each to the nearest gram. We then randomly selected 30 hatchlings (14–16 male and female) from each clutch for inclusion in the study (*n*=60). We implanted each study animal with a uniquely coded passive integrated transponder tag (PIT tag; Biomark, Boise, ID, USA) in compliance with our Florida Fish and Wildlife Conservation Commission permits (see ‘Permits and approvals’ section below). We housed hatchlings individually in ventilated, 6-quart plastic tubs lined with paper towels and provided water *ad libitum*. We maintained animals for 12 weeks after hatching in a climate-controlled room (23°C, 50–67% humidity) with windows providing ambient light and supplied each with a heat source, creating a thermal gradient of 27–33°C within each tub. At the conclusion of the study, we euthanized all animals using a 0.22 caliber Cash^®^ Special captive bolt gun (QC Supply, Schuyler, NE, USA) following methods approved by the American Veterinary Medical Association to minimize suffering and distress (Leary et al., 2013).

### Study design

We randomly assigned 30 hatchlings (seven to eight of each sex from each clutch) to one of two feeding treatment groups; a low food availability treatment (Low; presented one frozen/thawed mouse every 3 weeks) or a high food availability treatment (High; presented one frozen/thawed mouse weekly). Beginning the fourth week after their hatching date, we presented each study animal with an approximately 9 g frozen/thawed mouse at intervals listed above by feeding treatment. Over the course of the 12 weeks after hatching, study animals were either offered a total of three mice (Low) or nine mice (High). The feeding frequencies of wild Burmese python hatchlings are unknown, so we chose these feeding intervals to ensure that the High treatment had significantly more feeding opportunities. A 3 week by 1 week feeding design has been used by other researchers to evaluate snake growth in response to varied food availability (Taylor and Denardo, 2005).

We divided our study into two phases characterized by hatchling feeding behavior. We classified the first 1–4 weeks (1–28 days) post-hatching as the yolk-absorption phase (yolk phase). During this phase pythons are purportedly sustained by absorbing their internal yolk stores (e.g. Madsen and Shine, 2002; Shine et al., 1997). We verified this asserted anorexia by offering mice to the first 30 study animals available to us during the yolk phase prior to the fourth week after their hatching date. Fifteen of these hatchlings were from the High treatment and 15 from the Low treatment, 14 were females and 16 males. The eating phase began at the end of week 4 and lasted through the end of the study at 12 weeks post-hatching (i.e. 29–84 days since hatching). We constrained the study to 12 weeks because postemergence growth rates of hatchling Burmese pythons are some of the fastest documented in snakes and decline with age (Reed and Rodda, 2009). Each week we recorded SVL and weight for each study animal 2–3 days prior to any feeding events and recorded feeding propensity (i.e. did or did not feed). We also recorded feeding behavior (bold=aggressive/immediately attempted to consume prey; neutral=neither aggressive nor shy; or shy=retreated from prey) at 10 weeks post-hatching, the final time that all the hatchlings would be offered food and so could be compared.

### Data analyses

We defined BCI as the residuals of log-log transformed weights by SVLs (BCI; Falk et al., 2017). To calculate morphometric growth rates by day (Δmorph d^−1^, i.e. ΔBCI d^−1^, ΔSVL d^−1^, and Δweight d^−1^), we used a bivariate regression of the measurement of interest (BCI, length, or weight) by days since hatching and subsequently calculated each study animal's slope by fitting individual linear regression lines. For total changes in morphometric measurements (total Δmorph, i.e. total ΔBCI, total ΔSVL, and total Δweight), we used each study animal's initial measurements at week 1 subtracted from that individual's final measurements at week 12. To evaluate the rate of morphometric change per day (growth rates in Δmorph d^−1^) and by behavioral phase, we used each study animal's difference in measurements between the number of days or weeks of interest. For parametric tests, we checked that the data met model assumptions for normality and equal variances.

Because hatchling growth is directly related to the amount of food consumed, we used one-way analyses of variances (ANOVAs) to investigate individual effects of feeding treatment, clutch, and sex on total Δmorph or total grams eaten from week 1 to week 12. We analyzed combined effects with Standard Least Squares mixed effect leverage models. We examined morphological changes between fixed effect groups (feeding treatment, clutch, sex, or their interaction terms) over time (e.g. repeated measurements of individuals by day, week, or behavioral phase) using repeated measures analyses with the restricted maximum likelihood (REML) method fit for linear mixed models as appropriate for analyzing pre-maturation growth (e.g. Quince et al., 2008). For these models we used BCI, SVL, and weight as response variables and feeding treatment, clutch, sex, day, week, and phase as fixed effects with animal ID as a random effect to account for repeated measures. We followed significant model tests with Least Squares Means Tukey–Kramer post-hoc pairwise comparisons or Student's *t*-tests to detect significant differences between fixed effect groups. We summarized feeding propensity (total number of times hatchlings ate when offered food over the course of the project) and tested by feeding treatment, clutch, and sex using ANOVA for continuous variables and tested categorical feeding behavior differences using Pearson Chi Squared contingency analyses.

All statistical analyses were carried out using JMP^®^ statistical software (version 14, 2018 SAS Institute Inc.).

### Permits and approvals

This work was conducted in accordance with Florida Fish and Wildlife Conservation Commission permits EXOT-15-16, EXOT-15-34, EXOT-15-35, and was approved by the USGS Fort Collins Science Center Institutional Animal Care and Use Committee (Protocol Number: 2015-09).

## Supplementary Material

Supplementary information
