# Supplementary material for: Clutch may predict growth of hatchling Burmese pythons better than food availability or sex
Source: Biol Open. 2021 Nov 19;10(11):bio058739. doi: 10.1242/bio.058739 (PMC8609237; doi:10.1242/bio.058739)
Supplement: Supplementary information [file biolopen-10-058739-s1.pdf]

Biology Open (2021): doi:10.1242/bio.058739: Supplementary information

Table S1. Average body condition indices (BCI), snout-vent lengths (SVL), and weights of hatchling pythons (*Python bivittatus* Kuhl) in each feeding treatment (Tx) by clutch (C1 and C2). Week 1 initial measurements are above, week 12 final measurements are below. Standard error for each mean (s.e.m.) is presented in parentheticals. Only the hatchling mean morphometric comparisons between clutches were different, as indicated in the statistical reports column (Stat). There were no differences in hatchling morphometric means between feeding treatments, summarized in the italicized bottom line of each morph section (all P>0.4; not shown).

		n	High Tx	(s.e.m.)	*Stat*	n	Low Tx	(s.e.m.)	*Stat*
**Week 1 (Initial)**									
**BCI**	C1	15	0.13	(0.01)	$F_{1,26}=114.9894$,	15	0.14	(0.01)	$F_{1,27}=97.8930$,
	C2	13	0.03	(0.01)	$P<0.0001$	14	0.02	(0.01)	$P<0.0001$
Mean BCI		*28*	*0.08*	*(0.01)*		*29*	*0.08*	*(0.01)*	
**SVL (cm)**	C1	15	62.6	(0.48)	$F_{1,26}=10.9638$,	15	62.7	(0.48)	$F_{1,28}=8.6226$,
	C2	13	60.3	(0.52)	$P=0.0027$	15	60.7	(0.48)	$P=0.0066$
Mean SVL		*28*	*61.5*	*(0.40)*		*30*	*61.7*	*(0.39)*	
**Weight (g)**	C1	15	156.9	(1.81)	$F_{1,26}=244.6568$,	15	160.1	(1.81)	$F_{1,27}=231.6355$,
	C2	13	117.8	(1.94)	$P<0.0001$	14	117.6	(1.87)	$P<0.0001$
Mean Weight		*28*	*138.7*	*(4.13)*		*29*	*139.6*	*(4.06)*	
**Week 12 (Final)**									
**BCI**	C1	15	0.01	(0.02)	$F_{1,26}=10.8189$,	15	-0.01	(0.01)	$F_{1,28}=19.0767$,
	C2	13	-0.07	(0.02)	$P=0.0029$	15	-0.09	(0.01)	$P=0.0002$
Mean BCI		*28*	*-0.03*	*(0.01)*		*30*	*-0.05*	*(0.01)*	
**SVL (cm)**	C1	15	77.4	(0.67)	$F_{1,26}=54.6739$,	15	77.2	(0.67)	$F_{1,28}=133.9678$,
	C2	13	68.5	(0.72)	$P<0.0001$	15	69.1	(0.67)	$P<0.0001$
Mean SVL		*28*	*73.3*	*(0.95)*		*30*	*73.1*	*(0.91)*	
**Weight (g)**	C1	15	176.4	(6.59)	$F_{1,26}=25.3846$,	15	170.1	(6.59)	$F_{1,28}=58.7193$,
	C2	13	119.5	(7.08)	$P<0.0001$	15	115.5	(6.59)	$P<0.0001$
Mean Weight		*28*	*150.0*	*(7.14)*		*30*	*142.8*	*(6.90)*	

Biology Open • Supplementary information
